# Large Yellow Croaker Roe Protein Isolates/Gellan Gum Hydrogels Improve the Alleviating Effect of Curcumin on DSS-Induced Colitis

**DOI:** 10.3390/foods14111921

**Published:** 2025-05-28

**Authors:** Yi-Nan Du, Yi-Xu Wang, Jia-Nan Yan, Qian Zhang, Yu-Qiao Wang, Jiao Jia, Hai-Tao Wu

**Affiliations:** SKL of Marine Food Processing & Safety Control, National Engineering Research Center of Seafood, Collaborative Innovation Center of Seafood Deep Processing, Key Laboratory of Aquatic Product Processing and Quality Control, School of Food Science and Technology, Dalian Polytechnic University, Dalian 116034, China; dyn7381@163.com (Y.-N.D.); 13234215787@163.com (Y.-X.W.); yjn3vv@163.com (J.-N.Y.); zhangqian7386@163.com (Q.Z.); wangyuqiao1231@163.com (Y.-Q.W.); tbz426304@163.com (J.J.)

**Keywords:** large yellow croaker (*Pseudosciaena crocea*), roe, protein isolates, gellan gum, curcumin, ulcerative colitis

## Abstract

In this study, large yellow croaker (*Pseudosciaena crocea*) protein isolates/gellan gum (PG) binary hydrogels with dense microstructure were used for embedding and delivery curcumin (Cur). The colitis-relieving effects of PG-Cur were further investigated using the dextran sulfate sodium (DSS)-induced ulcerative colitis (UC) mouse model. Following PG-Cur treatment, weight loss, diarrhea, and shortening of the colon were significantly alleviated. Compared with the free Cur group, weight loss and colon length in the PG-Cur group increased about 1.05- and 1.12-fold. IL-1β, IL-6, TNF-α, and IL-10 levels in PG-Cur group were not significantly different from those of the normal mice, and the MPO and iNOS activities of the PG-Cur group were 29% and 20% lower than those in the Cur group, respectively. Moreover, fecal microbiota analysis of mice revealed that PG-Cur effectively restored gut dysbiosis in DSS-induced colitis, enriching beneficial bacteria while reducing harmful ones. Overall, the PG hydrogels have the potential to serve as carriers for oral curcumin formulations aimed at alleviating UC.

## 1. Introduction

Inflammatory bowel disease (IBD), a prevalent chronic condition driven by immune dysregulation, is chiefly characterized by ulcerative colitis (UC) and Crohn’s disease [1]. Recently, the prevalence of UC has sharply increased in Asia and economically underdeveloped regions. Current therapeutic strategies for UC mainly involve medications such as aminosalicylates, corticosteroids, immunosuppressants, and biologics, but these drugs cause certain adverse reactions, including diarrhea and osteoporosis [2]. Therefore, there is a growing interest in exploring natural and effective strategies for the alleviation of UC.

Curcumin (Cur), a natural polyphenolic compound, has shown significant potential in alleviating colitis due to its antioxidant, anti-inflammatory, and free radical-scavenging ability [3]. Curcumin alleviates inflammation by downregulating mediators like iNOS, COX-2, and MCP-1, thereby limiting nitric oxide and prostaglandin synthesis and modulating various signaling pathways [4,5]. However, the clinical use of Cur is hindered by its low solubility, fast metabolic rate, and limited bioavailability, which restrict its effectiveness in the colon when administered orally. To address these challenges, various encapsulation systems, including nanoparticles, emulsions, liposomes, and hydrogels, have been developed to improve the delivery and stability of Cur [6,7]. Previous studies have shown that Cur-loaded lactoferrin nanoparticles [8], Cur-loaded liposomes in pectin–chitosan hydrogels [9], and Cur-loaded pea protein high internal phase emulsions [10] demonstrated enhanced effectiveness in relieving UC symptoms relative to unencapsulated Cur. Among different delivery systems, hydrogels also show particular promise in improving the bioavailability of bioactive materials due to their unique network. Hydrogels are three-dimensional network materials with high water content and porous structures that enable active substances to be efficiently loaded [11,12]. For example, previous studies have prepared hydrogels for the encapsulation and delivery of active substances, including casein/chitosan gels for quercetin [13] and bovine serum albumin/citrus peel pectin gels for vitamin C [14]. Several hydrogel-based Cur-loaded systems were constructed, such as whey protein isolate–chitosan [15] and propylene glycol alginate/zein hydrogels [16]. These studies indicated that hydrogel systems could deliver more Cur to the colon, but these studies were mainly evaluated using in vitro experiments. Studies investigating the use of Cur-loaded hydrogels to exert active effects in the colon are limited. Yan et al. [17] found that compared to free Cur, Cur encapsulated within scallop hydrolysates/κ-carrageenan hydrogels exerted more pronounced effects in reinforcing the intestinal barrier, mitigating oxidative stress, and modulating the gut microbiota in a UC mouse model. However, the preventing effect on colitis of other Cur-loaded hydrogel system is still very limited.

The large yellow croaker (*Pseudosciaena crocea*) is an important aquaculture species in China, valued for both its economic and nutritional qualities. During its processing, roes are generated as byproducts and serve as a rich source of protein. Previous studies have found that the protein isolates from *P. crocea* roes (pcRPIs) contain higher level of essential amino acids than soy protein isolates, showing better oil holding capacity and emulsifying abilities [18]. The pcRPIs exhibited binding effect with polyphenols, including EGCG [19] and Cur [20]. The pcRPIs mainly bind with Cur by hydrogen bonds, van der Waals interactions, and hydrophobic interactions, and the cold-set pcRPI gels showed certain protective effects on Cur in the resistant gastrointestinal environment [20]. Furthermore, our previous research demonstrated that pcRPIs and gellan gum (GG) have a good synergistic gelation effect, and the pcRPI/GG binary gels exhibited excellent encapsulation and delivery capabilities for Cur. The composite hydrogels maintained their structure during simulated gastrointestinal digestion and protected approximately 76.6% of Cur from release, which allowed a significant portion of Cur to be delivered to the colon [21]. However, the previous study was mainly conducted using in vitro digestion models, and more in-depth in vivo animal experiments have not yet been carried out. The effects of Cur-loaded pcRPI/GG hydrogels on alleviating UC also have not been explored.

In this study, we prepared Cur-loaded pcRPI/GG hydrogels (PG-Cur) and evaluated the microstructure properties of different hydrogels. Then, the effects of PG-Cur on DSS-induced colitis in mice were also analyzed. Specifically, disease active index (DAI) variation; colon variation; the expression level of myeloperoxidase (MPO), inducible nitric oxide synthase (iNOS), and inflammatory factors; as well as gut microbiota composition were evaluated.

## 2. Materials and Methods

### 2.1. Materials and Chemicals

*P. crocea* roes were provided by Qingdao Yujie Group Co. Ltd. (Qingdao, China). Curcumin was obtained from Sigma-Aldrich (St. Louis, MO, USA), while dextran sulfate sodium (DSS) was sourced from Yeasen Biotechnology Co., Ltd. (Shanghai, China). Gellan gum (GG) and glucono-δ-lactone (GDL) were purchased from Macklin Biochemical Co., Ltd. (Shanghai, China). The bicinchoninic acid (BCA) protein assay kits were provided by Beijing Solarbio Technology Co., Ltd. (Beijing, China). Assay kits for MPO and iNOS were obtained from Nanjing Jiancheng Bioengineering Institute (Nanjing, China). ELISA kits for cytokines including TNF-α, IL-1β, IL-6, and IL-10 were acquired from Shanghai Enzyme-linked Biotechnology Co., Ltd. (Shanghai, China). All reagents used in this study were of analytical grade.

The *P. crocea* protein isolate was prepared according to our previous studies [18]. Briefly, 10 g of freeze-dried *P. crocea* roe powders were suspended in 200 mL of 0.6 M NaCl solution and stirred continuously for 2 h at 300 rpm. The mixture was then centrifuged at 8000 rpm for 15 min. The supernatant was carefully collected, and the solid residue was subjected to a second extraction using the same procedure. The combined supernatants were dialyzed and lyophilized to obtain pcRPIs.

### 2.2. Preparation of Cur-Loaded PG Hydrogels

As presented by Du et al. [21] and Yan et al. [22], pcRPIs were initially dispersed in deionized water at a final concentration of 31.1 mg/mL and fully hydrated at 4 °C for 12 h. After hydration, the pH of the solution was carefully adjusted to 7.0 using 1 mol/L NaOH under continuous stirring at 300 rpm for 3 h. The resulting mixture was then subjected to thermal treatment at 80 °C for 1 h to induce protein denaturation, after which GG powder was incorporated at a concentration of 2.2 mg/mL. Upon cooling, Cur was first fully dissolved in absolute ethanol with the content of 160 mg/m. To ensure a daily Cur intake of 80 mg/kg body weight for mice, the final concentration of Cur in the system was adjusted to 16 mg/mL, and then 0.2% GDL was added into the mixture. The Cur loaded PG gel (PG-Cur) was stored at 4 °C until further analyses. In addition, a 16 mg/mL Cur suspension in deionized water and pcRPI/GG gels devoid of Cur (PG) served as controls.

### 2.3. Cryo-Scanning Electron Microscopy (Cryo-SEM)

Gel samples were characterized using Cryo-SEM on a Hitachi SU8010 instrument (Hitachi Co., Ltd., Tokyo, Japan). Samples were first plunge-frozen in liquid nitrogen and then transferred into a PP3010T cryo-preparation system (Quorum Technologies, Laughton, UK) for freeze-fracture, sublimation, and platinum sputter coating. Imaging was conducted at 10 kV accelerating voltage, with micrographs recorded at 2000× magnification.

### 2.4. Animals

Six-week-old male BALB/c mice (18–22 g, SPF grade) were procured from Liaoning Changsheng Biotechnology Co., Ltd. (Benxi, China). Animals were maintained in the SPF facility of the Animal Experiment Center at Dalian Polytechnic University (permit no. SYKX2017-0005) under controlled conditions (25 ± 2 °C, 60 ± 5% relative humidity, 12 h light/12 h dark cycle). The mice had ad libitum access to deionized water and standard feed. All experimental procedures received approval from the Institutional Animal Care and Use Committee of Dalian Polytechnic University (animal welfare and ethics approval number: DLPU2022004) and complied with ethical regulations.

### 2.5. Dietary Intervention and DSS-Induced Ulcerative Colitis in Mice

**Figure 1:**
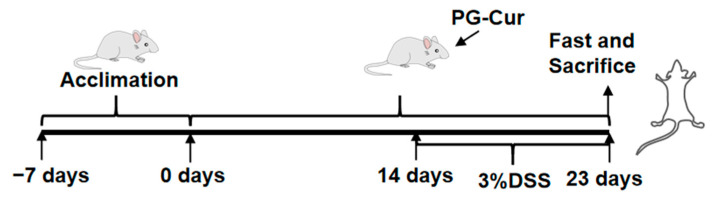
Schematic clarification of the experimental procedure.

Ulcerative colitis was induced using dextran sulfate sodium (DSS) following the protocol of Cao et al. [23]. Animals were randomly assigned to experimental groups by the experimenter using a simple randomization approach to ensure balanced group sizes. The allocation was performed prior to the start of treatment, and all outcome assessments were conducted without knowledge of the group assignments. Briefly, sixty mice were randomly allocated into five experimental cohorts (*n* = 12 each): (1) Control group; (2) DSS-treated model group (DSS); (3) PG intervention group (PG + DSS); (4) Free Cur intervention group (Cur + DSS); and (5) PG-Cur intervention group (PG-Cur + DSS). As shown in Figure 1, mice were acclimatized for 7 days with standard feeding and free access to deionized water. Starting on day 8, PG, free Cur, or PG-Cur were intragastrically administered with continuing standard feeding and providing free access to deionized water. Mice in each treatment cohort received a daily oral gavage of 100 μL of their designated sample; Control and DSS-only groups were similarly administered 100 μL of deionized water. From day 22 to day 31 (9 days), the DSS model group, PG + DSS, Cur + DSS, and PG-Cur + DSS groups were allowed to freely ingest DSS solution (3%, *w*/*v*), with the solution being replaced every two days. The Control group continued to have free access to deionized water. At the end of the experiment, mice were humanely euthanized. Blood was collected for serum isolation; colon tissues and intestinal contents were collected and stored at −80 °C for subsequent analyses. A separate segment of colon was fixed in 4% paraformaldehyde for hematoxylin and eosin (H&E) histological assessment.

### 2.6. Mouse DAI Assessment

The DAI was quantified following previous studies, based on scoring body weight loss, stool consistency, and the presence of fecal blood [24]. Body weight loss was assessed on a four-point scale. Specifically, reductions of <5%, 5–10%, 10–20%, and >20% were assigned scores of 1, 2, 3, and 4, respectively. Stool form was evaluated as 0 for normal, 2 for loose stools, and 4 for diarrhea. Fecal bleeding was graded 0 for absent, 2 for occult or minor bleeding, and 4 for overt hemorrhage.

### 2.7. Colonic Histopathological Analysis

Colon samples were subjected to standard histological processing, including dehydration, clearing, and embedding in paraffin. Tissue blocks were sectioned and stained with H&E. Histological examination was subsequently conducted using light microscopy (Nikon Eclipse Ti-S, Tokyo, Japan) to assess pathological changes.

### 2.8. Enzymatic Activities of Myeloperoxidase (MPO) and Inducible Nitric Oxide Synthase (iNOS)

Colonic tissue samples were homogenized with physiological saline for 2 min to obtain a 10% tissue homogenate. The enzyme activities of MPO and iNOS, as well as the protein content, were determined according to the instructions provided with the MPO, iNOS, and BCA assay kits.

### 2.9. TNF-α, IL-1β, IL-6, and IL-10 Levels

After euthanasia, blood samples were collected and left undisturbed for 2 h. After centrifugation at 2500× *g* for 10 min at 4 °C, serum was harvested from the samples. Concentrations of TNF-α, IL-1β, IL-6, and IL-10 were then quantified using ELISA kits in accordance with the manufacturers’ protocols, and the results were calculated accordingly.

### 2.10. Analysis of Gut Microbiota Composition

#### 2.10.1. DNA Extraction and PCR Amplification

Microbial genomic DNA was isolated from colonic fecal samples using the E.Z.N.A.^®^ Soil DNA Kit (Omega Bio-tek, Norcross, GA, USA) following the supplier’s guidelines. DNA integrity was verified by electrophoresis on a 1% agarose gel, and its concentration and purity were subsequently determined. PCR amplification of the V3–V4 hypervariable segment of the bacterial 16S rRNA gene was carried out using primers 338F (ACTCCTACGGGAGGCAGCAG) and 806R (GGACTACHVGGGTWTCTAAT).

#### 2.10.2. Illumina MiSeq Sequencing

High-throughput sequencing was performed following previously described methods [23]. PCR amplicons were separated on a 2% agarose gel and subsequently purified using the AxyPrep DNA Gel Extraction Kit (Axygen Biosciences, Union City, CA, USA). The purified products were then quantified using fluorescence-based methods. Library construction was performed using the NEXTFLEX Rapid DNA-Seq Kit, followed by high-throughput sequencing for downstream microbial community analysis.

### 2.11. Statistical Analysis

All data were examined at least in three times, and all results are presented as mean ± standard deviation. The data were analyzed based on one-way analysis of variance (ANOVA) coupled with Tukey’s multiple comparison test by using SPSS software version 11.5, with differences considered significant at *p* < 0.05. Microbial community analysis, including taxonomic abundance profiling, heatmap visualization, and LEfSe differential analysis, were conducted using the online analysis platform provided by Majorbio Biomedical Technology Co., Ltd. (Shanghai, China). (https://www.majorbio.com, accessed on 1 December 2022).

## 3. Results and Discussion

### 3.1. Visual Appearance and Microstructural Properties of PG and PG-Cur

**Figure 2:**
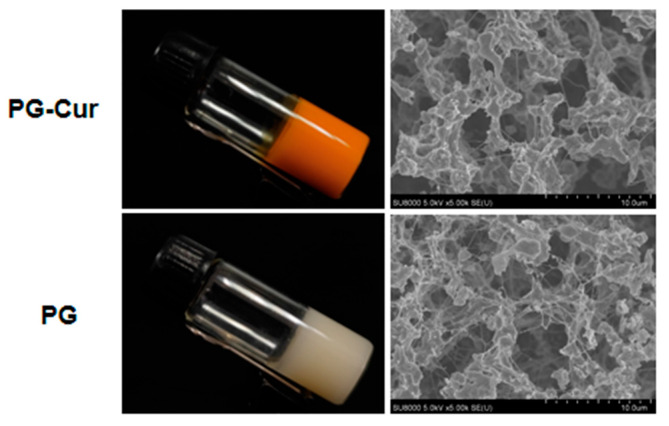
Visual appearance and Cryo-SEM micrographs of PG and PG-Cur gels.

Previous studies demonstrated that GG could significantly enhance the gelation characteristics of pcRPI gels, facilitating the development of a novel dual-network hydrogel composed of pcRPIs and GG. This composite hydrogel exhibited superior encapsulation capacity and protective performance for Cur, effectively minimizing its release during gastrointestinal transit. As a result, a greater proportion of Cur was preserved and preferentially delivered to the colon, where it could exert its biological effects [21]. To this point, an animal experimental model was further used to evaluate the effect of pG-Cur on colitis. As shown in Figure 2, the PG-Cur gel exhibited a uniform bright tangerine color, indicating efficient incorporation and uniform distribution of Cur within the gel. The Cryo-SEM results showed that both PG and PG-Cur hydrogels feature densely packed protein aggregates embedded within an interwoven network. These results were similar to our previous studies [21], and Cur also improved the crosslinking between PG-Cur gels. Therefore, PG-Cur hydrogels with a homogeneous network could serve as efficient colon-targeted carriers for Cur, facilitating its localized release and therapeutic action.

### 3.2. Effect of PG-Cur on Body Weight, DAI, and Colon Length in a DSS-Induced UC Mouse Model

**Figure 3:**
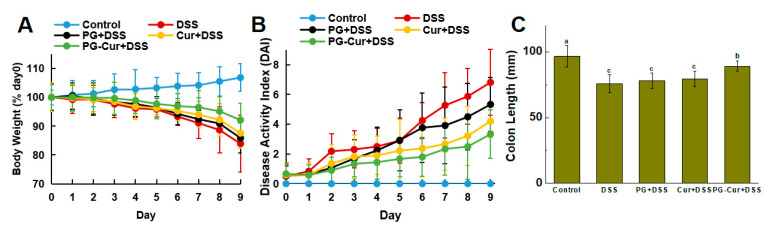

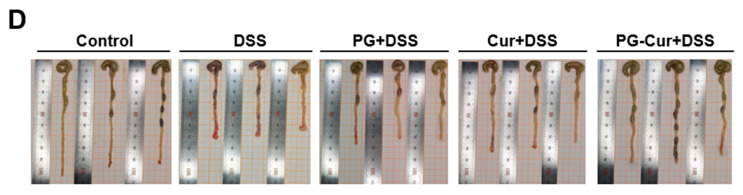
The changes in body weight (**A**), DAI scores, (**B**) colon length (**C**) and colon tissue appearance (**D**) in different groups. The various letters (a–c) in (**C**) indicate significant differences (*p* < 0.05) among each group.

To assess the therapeutic effects of the PG-Cur gels in a DSS-induced UC mouse model, the body weight changes and DAI scores of mice were further evaluated. As shown in Figure 3A, after 9 days of DSS treatment, the Control group exhibited a 7% increase in body weight, whereas the DSS group and PG group displayed significant 16% and 14% reductions in body weight, respectively. Progressive weight gain in the control cohort is likely attributable to normal physiological development, aligning with previous reports of a 9% increase by Yan et al. [17] and a 7% rise observed by Luo et al. [2]. Many studies have also shown that after DSS induction, mice show a trend toward significant weight loss [25]. In contrast, the PG-Cur group only experienced an 8% weight loss, which was significantly lower than the 13% weight loss observed in the Cur group. These results indicated that DSS induced colitis and resulted in weight loss in mice, which was effectively alleviated by PG-Cur treatment. The DAI scores assess the severity of colitis based on clinical symptoms such as diarrhea, stool, and other conditions. Figure 3B shows that the DAI scores in the DSS-treated group rose markedly from day 3, reaching 6.5 by day 9. This result indicated that DSS treatment could cause UC in mice. Moreover, in the PG-Cur group, DAI scores remained significantly lower compared to the Cur and PG groups, with a final score of 3.3, which was closest to the Control group. These results indicate that the mouse colitis model induced by DSS was successful, which is comparable to the performance of the mouse colitis models obtained using methods such as 3% DSS induction for 7–9 days [17,26,27], 4% DSS induction for 7 days [28], as well as 5% DSS induction for 7 days [29]. Yan et al. [17] also showed that, compared to the free Cur group, the Cur-loaded scallop hydrolysates/κ-carrageenan hydrogels group exhibited an approximately 1.04-fold greater body weight loss, along with a 13% reduction in DAI scores. Zhang and Li [30] demonstrated that an eight-day treatment with a Cur-loaded biopolymeric nanocomposite significantly reduced DAI scores compared to the DSS-only group.

Moreover, after DSS induction, significant alterations were observed in the colon morphology of mice, including shortened colon length, thickened intestinal walls, congestion, and swelling. As shown in Figure 3C,D, in the Control group, the colon length was approximately 96.6 mm, with a smooth surface, while the DSS group exhibited a marked reduction in colon length, which decreased to around 75.7 mm. However, following PG-Cur treatment, the shortening of the colon was alleviated, with the final colon length reaching 89.0 mm. Although free Cur also showed some improvement in colon length, the degree of alleviation was modest, with the colon measuring approximately 79.3 mm, which was not significantly different from the model group. Overall, relative to the Cur group, the PG-Cur group exhibited approximately 1.05-fold greater body weight loss and a 1.12-fold increase in colon length. Furthermore, the PG group, lacking any active substances, did not exhibit any notable therapeutic effects, with a final colon length of 78 mm. Zhang et al. [31] prepared alginate/chitosan hydrogel (Cur@NMPs) for Cur delivery and also found that with Cur and Cur@NMPs treatment, the colon shortening of mice was delayed to varying degrees. In addition, the colon length of Cur@NMPs group mice most closely approached the normal value. The poor efficacy of free Cur stems from its very low oral bioavailability and rapid degradation in the gastrointestinal tract, which prevents sufficient amounts from reaching the colon. By contrast, the PG hydrogel forms a cage-like network that protects Cur in the stomach and small intestine but disassembles in the colon. Gellan gum also has interesting characteristics of pH-dependent tunable behavior and resistance to the activities of most human enzymes, which makes GG a promising prospect for the development of encapsulation systems for delivering active substances to specific parts of the gastrointestinal tract [32]. Furthermore, the interaction between GG and pcRPIs forms a more compact, dense microstructure, which enhances the carrier’s resistance to digestive enzymes and mechanical stresses [21]. Moreover, our previous studies investigated the interaction of pcRPIs with Cur using molecular docking analysis and found that van der Waals interactions, hydrophobic interactions, and hydrogen bonds were the main driving forces in the binding of pcRPIs with Cur [20]. Previous studies have also shown that the -OH functional groups in Cur can combine with GG through intermolecular interactions such as hydrogen bonds [33,34]. The interactions among pcRPIs, GG, and Cur facilitate the entrapment of Cur within the gel network, leading to delaying its release under gastrointestinal digestion conditions. Therefore, these results indicated that PG-Cur can effectively deliver Cur to the colon, where it exerts its anti-inflammatory and antioxidant properties, leading to significant alleviation of symptoms associated with UC.

### 3.3. Effect of PG-Cur on Colon Variations in the DSS-Stimulated UC Mouse Model

**Figure 4:**
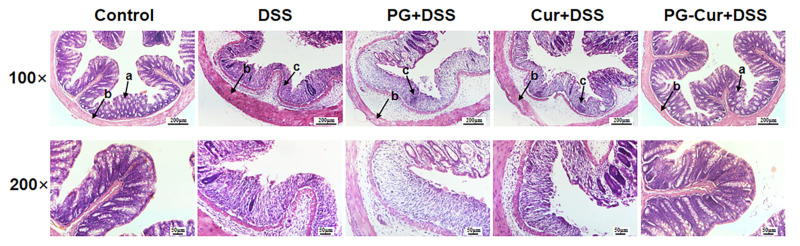
Histological images of colonic tissues from various treatment groups. DSS, dextran sulfate sodium-treated model group; PG + DSS, *P*. *crocea* roe protein isolates/gellan gum hydrogels intervention group; Cur + DSS, free curcumin intervention group; PG-Cur + DSS, curcumin loaded *P*. *crocea* roe protein isolates/gellan gum hydrogels intervention group. a, goblet cells; b, muscle layer; c, inflammatory cells.

Hematoxylin and eosin staining of colon tissue sections was performed to evaluate the histological effects of PG-Cur treatment on DSS-induced colitis. As shown in Figure 4, in the Control group, the mucosal epithelium structure of the colon was clear and intact, with well-organized glandular arrangements and no infiltration of inflammatory cells. In contrast, the model group induced by DSS exhibited significant pathological changes in the colonic tissue, and the colonic mucosal epithelial cells were seriously damaged, including partial glandular destruction, crypt distortion, incomplete mucosal integrity, loss of goblet cells, as well as infiltration of inflammatory cells. Similar results were observed in colonic tissues from other DSS-induced UC mice [24,35]. Following PG treatment alone, the colonic tissue displayed structural changes resembling those of the DSS group, indicating that PG did not offer significant protection. Moderate infiltration of inflammatory cells and a reduced number of goblet cells were observed in the colonic tissues of mice treated with Cur, with only slight alleviation of erosion and ulceration compared to the DSS group. Notably, PG-Cur treatment resulted in the lowest levels of inflammatory cell infiltration in both the mucosal and submucosal layers among all groups. The PG-Cur group exhibited no apparent ulceration, with well-preserved glandular structures and intact epithelium. These results indicate that PG-Cur effectively protected the colonic morphology of mice with DSS-induced inflammation.

### 3.4. Effect of PG-Cur on MPO and iNOS Activity in the DSS-Stimulated UC Mouse Model

**Figure 5:**
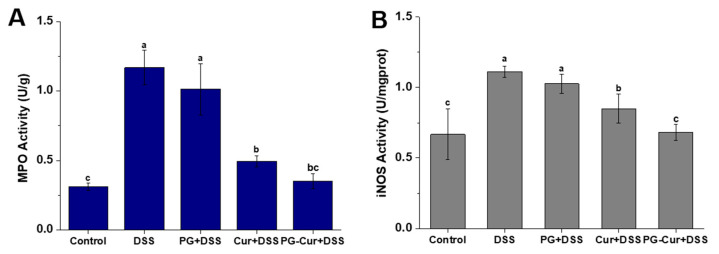
Effects of PG-Cur on MPO (**A**) and iNOS (**B**) activity. The various letters (a–c) indicate significant differences (*p* < 0.05) among each group.

MPO and iNOS are well-established markers of inflammation. MPO, an enzyme predominantly found in neutrophils, is a crucial indicator of neutrophil infiltration and the severity of inflammation, with increases in its activity and levels reflecting the extent of inflammatory damage [36]. As shown in Figure 5, the MPO and iNOS activities of the Control group were 0.31 U/g and 0.67 U/mgprot, respectively. These activities significantly increased in the DSS group, reaching 1.17 U/g and 1.11 U/mgprot, respectively. Elevated MPO and iNOS levels have been widely reported in DSS-induced colitis models and are positively associated with inflammatory severity and mucosal injury [37,38]. Notably, both Cur and PG-Cur effectively suppressed MPO and iNOS levels in the colons of mice, with PG-Cur demonstrating a more pronounced inhibitory effect. In the PG-Cur group, MPO and iNOS activities were reduced to 0.35 U/g and 0.68 U/mgprot, which were 29% and 20% lower than those in the Cur group, respectively. Inflammation exacerbates oxidative stress by stimulating reactive oxygen and nitrogen species (ROS/RNS) generation, a process in which MPO and iNOS play pivotal roles. Previous studies also demonstrated that Cur exerts inhibitory effects on the expression of MPO and iNOS in mice with colitis [39]. To this point, PG-Cur showed the ability to deliver Cur efficiently to the colon, and its anti-inflammatory action likely underpins its potent suppression of MPO and iNOS activity. These findings align with the DAI and histological changes observed in the colons of mice.

### 3.5. Effect of PG-Cur on Protein Levels of IL-6, IL-1β, TNF-α, and IL-10 in the DSS-Stimulated UC Mouse Model

**Figure 6:**
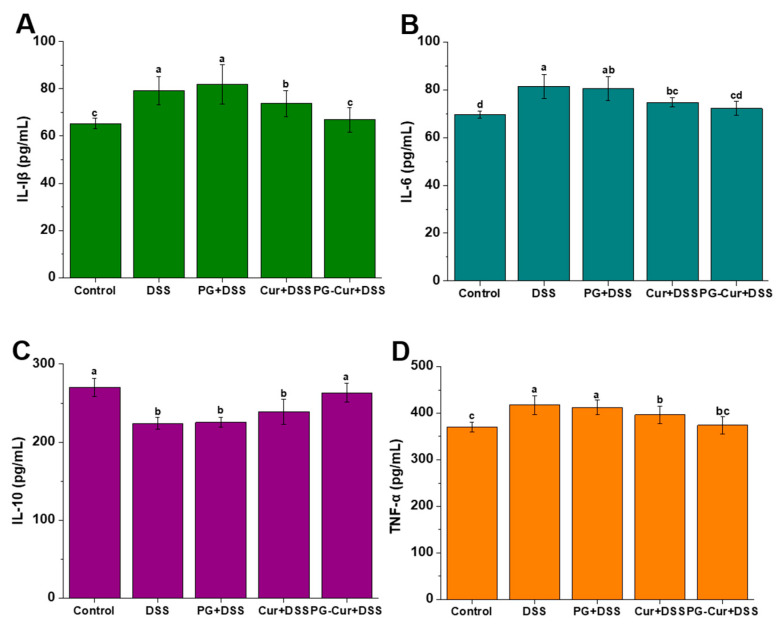
Effects of PG-Cur on the amount of inflammatory factors in mouse serum, including IL-1β changes (**A**), IL-6 changes (**B**), IL-10 changes (**C**) and TNF-α changes (**D**). The various letters (a–d) indicate significant differences (*p* < 0.05) among each group.

As shown in Figure 6, the contents of IL-1β, IL-6, TNF-α, and IL-10 in the Control group were 65.32 pg/mL, 69.66 pg/mL, 370.49 pg/mL, and 270.05 pg/mL, respectively. Compared to the Control group, the DSS group exhibited a significant upregulation in the levels of IL-1β, IL-6, and TNF-α, while IL-10 expression was notably downregulated. However, after intervention with PG-Cur, inflammatory responses were alleviated, with IL-1β, IL-6, and TNF-α levels reduced to 66.91 pg/mL, 72.26 pg/mL, and 374.23 pg/mL, respectively, while IL-10 levels increased to 263.21 pg/mL. These cytokine levels were statistically indistinguishable from those observed in the Control group (*p* > 0.05). The free Cur intervention also showed a reduction in pro-inflammatory cytokines, but the effect was significantly weaker than that of the PG-Cur group. Additionally, the PG group showed no notable impact on the regulation of either pro-inflammatory or anti-inflammatory cytokines. Hu et al. [40] also found that IL-6 and TNF-α levels decreased to levels similar to healthy controls after oral gavage of Cur-loaded heterogeneous double-membrane microgels, and the levels were also lower than those in the free Cur group.

The pathogenesis of UC is intricately linked to the overproduction of inflammatory mediators, which significantly exacerbate the inflammatory response and contribute to the progression of intestinal damage. Among these mediators, IL-6 plays a pivotal role as a pro-inflammatory cytokine, significantly contributing to UC inflammation. IL-6 mediates its activity by activating the STAT-3 signaling pathway and enhancing the expression of anti-apoptotic proteins, thereby facilitating CD4+ T cell accumulation and suppressing apoptotic processes [41]. Similarly, TNF-α is another central cytokine involved in the inflammatory cascade. TNF-α signaling orchestrates various pro-inflammatory actions, including the promotion of angiogenesis, necroptosis, and immune-mediated pathology. TNF-α also activates myosin light chain kinase, contributing to the disruption of the epithelial barrier and exacerbating immune pathology [42]. IL-10, a well-known anti-inflammatory cytokine, plays a protective role in UC by modulating immune responses [43]. Furthermore, IL-1β is another key mediator involved in the pathogenesis of UC, primarily acting through the activation of innate lymphoid cells and promoting the recruitment of granulocytes to the site of inflammation. IL-1β facilitates the early phase of innate immune responses, leading to an exacerbation of the inflammatory process and the subsequent recruitment of inflammatory cells to the affected tissues. These results suggest that Cur, when delivered via gel loading, exerts a pronounced anti-inflammatory effect in the DSS-induced UC mouse model.

### 3.6. Effect of PG-Cur on Microbial Composition in the DSS-Stimulated UC Mouse Model

#### 3.6.1. Microbial Composition Analysis

**Figure 7:**
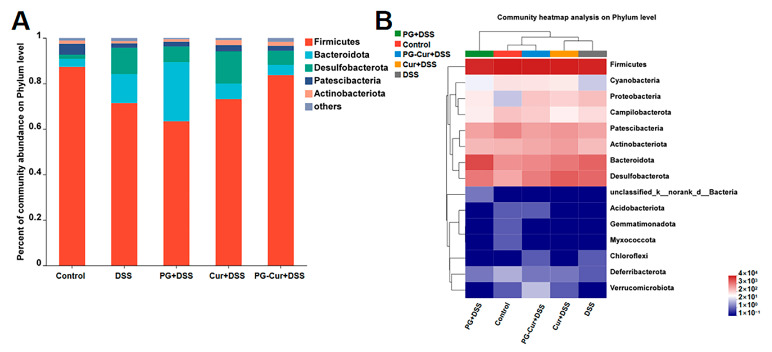
Gut microbiota composition of mice in different groups: (**A**): Histogram of percent of microbial abundance at the phylum level; (**B**): Microbial heatmap analysis at the phylum level.

As shown in Figure 7A, the dominant phyla of the Control group were *Firmicutes* (87.32%), *Patescibacteria* (4.83%), *Bacteroidetes* (3.51%), *Desulfobacterota* (1.84%), and *Actinobacterteriota* (1.32%). After DSS treatment, the gut microbiota underwent significant changes, with a marked decrease in *Firmicutes*, *Patescibacteria*, and *Actinobacterteriota* abundance, which dropped to 71.39%, 1.97%, and 0.97%, respectively. Conversely, the abundance of *Bacteroidetes* and *Desulfobacterota* increased to 12.72% and 11.58%, respectively. Intervention with PG or free Cur had minimal effects on the gut microbiota composition, with values similar to those of the DSS group. However, after PG-Cur intervention, the abundance of *Firmicutes*, *Patescibacteria*, and *Actinobacterteriota* increased to 83.64%, 22.23%, and 1.72%, respectively, while the abundance of *Bacteroidetes* and *Desulfobacterota* decreased to 4.52% and 6.21%, respectively, mitigating the adverse effects of DSS on the gut microbiota. Additionally, the *Firmicutes*/*Bacteroidetes* (F/B) ratio is considered an indicator of gut dysbiosis. The F/B ratio in the Control group was 24.88. After DSS treatment, this ratio significantly decreased to 5.61. However, following PG-Cur intervention, the F/B ratio partially recovered to 18.50. As reported, the intestinal microbiota of patients with colitis is significantly disturbed compared with normal people, and the F/B ratio was significantly reduced in colitis patients [44], which is similar to our studies. Previous studies by Li et al. and Yang et al. [45] also reported a reduction in the F/B ratio following DSS induction, which was reversed after intervention with either ginseng polysaccharides or *Sporisorium reilianum* polysaccharides. Recognized for its anti-inflammatory activity, *Actinobacteriota* contributes significantly to the regulation of gut inflammation. In this study, we observed that *Actinobacteriota* abundance decreased after DSS induction, but was restored to normal levels following PG-Cur intervention. Li et al. [46] also demonstrated that arabinose supplementation promoted the growth of *Actinobacteriota* in DSS-induced colitis models.

In addition, Figure 7B shows a species richness heatmap of the mouse gut microbiota at the phylum level, with color changes representing differences in relative abundance. The results are consistent with those in Figure 7A. After DSS induction, the abundance of *Cyanobacteria*, *Acidobacteriota*, *Gemmatimonadota*, *Myxococcota*, *Deferribacterota*, and *Verrucomicrobiota* decreased, while *Proteobacteria*, *Bacteroidota*, *Desulfobacterota*, and *Chloroflexi* increased. These changes indicated the development of colitis and microbial dysbiosis induced by DSS. The individual PG and Cur treatments had limited effects on modulating the gut microbiota. However, after PG-Cur intervention, the abundances of *Cyanobacteria*, *Acidobacteriota*, *Bacteroidota*, and *Desulfobacterota* were restored to levels similar to those of the Control group. Additionally, the abundance of *Verrucomicrobiota* increased and slightly exceeded that of the Control group.

#### 3.6.2. Heatmap Analysis

**Figure 8:**
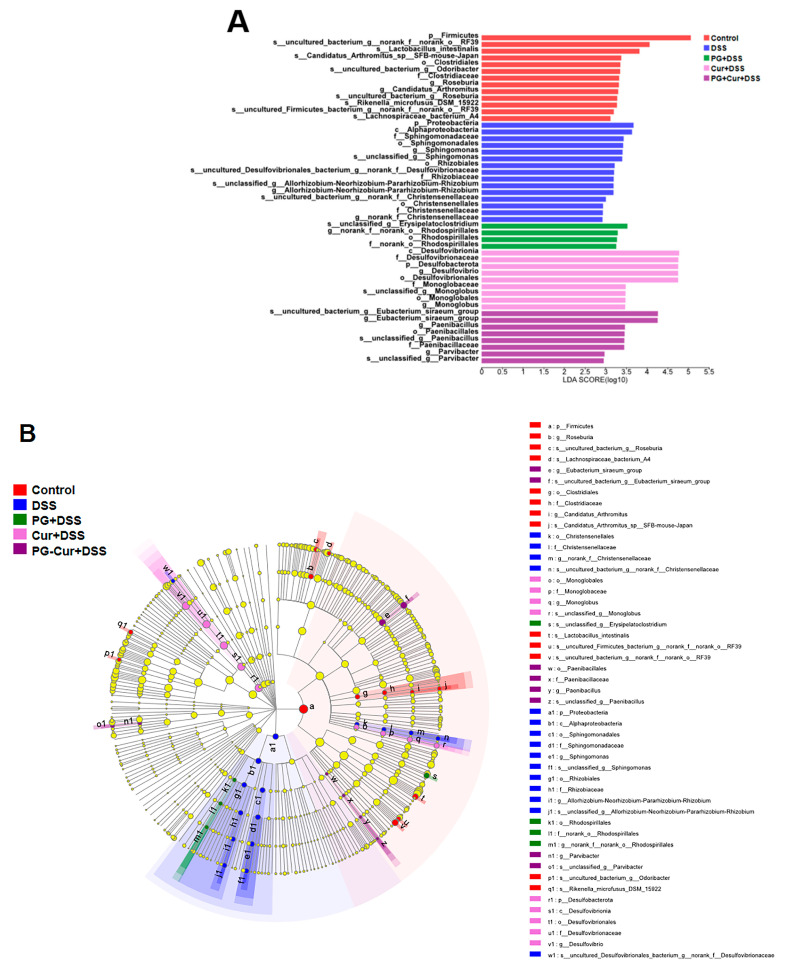
LEfSe species difference analysis of mice in different groups: (**A**): Multistage species hierarchical tree map; (**B**): Linear discriminant analysis (LDA) results.

The LEfSe algorithm was applied to identify significant microbial community or species differences among groups based on their linear discriminant analysis (LDA) scores. As shown in Figure 8, the results were visualized through a bar chart and a phylogenetic tree. The phylogenetic tree was used to illustrate the microbial community differences between groups at various taxonomic levels. Each circle in the tree represents a taxonomic level, with different colors indicating the presence of significantly different species between groups. Yellow circles denote species with no significant differences across the groups. Specifically, in the Control group, there was a significant enrichment in *Firmicutes* at the phylum level, RF39 at the order level, and *Lactobacillus* intestinalis at the genus level. However, following DSS-induced colitis, a marked shift in the microbial community was observed. At the phylum level, *Proteobacteria* was significantly enriched, while at the class level, *Alphaproteobacteria* predominated. Moreover, after PG intervention, the microbial community in the mouse gut showed a significant enrichment of *Erysipelatoclostridium* at the genus level and *Rhodospirillales* at the order level. Free Cur intervention led to a notable enrichment of *Desulfovibrionia* at the class level, *Desulfovibrionaceae* at the family level, and *Desulfobacterota* at the phylum level. The PG-Cur intervention showed a significant increase in *Eubacterium siraeum*, *Paenibacillus*, *Paenibacillales*, and *Paenibacillaceae* at the genus, order, and family levels, respectively. *Lactobacillus* intestinalis, a well-known beneficial bacterium, was notably enriched in the gut microbiota of normal mice. This bacterium is commonly regarded for its role in maintaining gut homeostasis and preventing pathogenic overgrowth [47]. *Proteobacteria*, is a pro-inflammatory bacterium, and previous reports have also demonstrated that after induction by DSS, the mouse gut is enriched with *Proteobacteria*, which drive pro-inflammatory changes in the gut and alter the gut microbiota [48,49]. *Desulfobacterota*, a pathogenic group that promotes the release of inflammatory cytokines and worsens colitis [50], showed enrichment in the free Cur group. This results suggest that free Cur might exert a relatively weaker effect in regulating gut microbiota, allowing the persistence of harmful microflora. Furthermore, *Paenibacillus*, a beneficial genus found in the rumen of animals, plays a key role in amino acid metabolism, reducing nitrosamine toxicity and methane emissions, while also contributing to the reduction of intestinal damage [51,52]. In this study, *Paenibacillus* was found to be enriched in the PG-Cur group. This suggests that PG-Cur may facilitate the proliferation of beneficial microbial taxa, including *Eubacterium* and *Paenibacillus*, which have been associated with positive impacts on gut health and immunity. Therefore, PG-Cur intervention can restore the dysbiosis induced by DSS, helping maintain a healthy gut microbiota balance and counteracting inflammatory responses.

## 4. Conclusions

DSS-induced colitis in mice led to notable symptoms such as diarrhea, shortened colon, and increased DAI scores, with histological damage to the colon observed using hematoxylin and eosin staining. Both free Cur and PG-Cur exhibited varying degrees of colitis relief, with PG-Cur showing superior efficacy. PG-Cur significantly inhibited MPO and iNOS levels, bringing them to levels comparable to the Control group. Additionally, PG-Cur effectively modulated the pro-inflammatory cytokines IL-1β, IL-6, and TNF-α, while upregulating the anti-inflammatory cytokine IL-10. For microbial composition results, DSS treatment led to an imbalance of gut microbiota and enrichment of harmful bacteria. However, PG-Cur intervention restored the microbiota composition, enriching beneficial bacteria and promoting gut homeostasis. These findings indicated that PG-Cur can serve as effective functional agents for alleviating colitis and regulating intestinal inflammation and dysbiosis.

## Data Availability

The original contributions presented in the study are included in the article; further inquiries can be directed to the corresponding author.

## Abbreviations

pcRPIs	*Pseudosciaena crocea* roe protein isolates
GG	gellan gum
Cur	curcumin
GDL	glucono-δ-lactone
PG	*Pseudosciaena crocea* roe protein isolates/gellan gum hydrogels
DSS	dextran sulfate sodium
UC	ulcerative colitis
DAI	disease activity index
LDA	linear discriminant analysis

## References

[1] Tian M., Li D., Ma C., Feng Y., Hu X., Chen F. (2021). Barley leaf insoluble dietary fiber alleviated dextran sulfate sodium-induced mice colitis by modulating gut microbiota. Nutrients.

[2] Luo R., Lin M., Zhang C., Shi J., Zhang S., Chen Q., Hu Y., Zhang M., Zhang J., Gao F. (2020). Genipin-crosslinked human serum albumin coating using a tannic acid layer for enhanced oral administration of curcumin in the treatment of ulcerative colitis. Food Chem..

[3] Goulart R.D.A., Barbalho S.M., Lima V.M., Souza G.A.D., Matias J.N., Araújo A.C., Rubira C.J., Buchaim R.L., Buchaim D.V., de Carvalho A.C.A. (2021). Effects of the use of curcumin on ulcerative colitis and crohn’s disease: A systematic review. J. Med. Food.

[4] Wang Y., Li Y., He L., Mao B., Chen S., Martinez V., Guo X., Shen X., Liu B., Li C. (2021). Commensal flora triggered target anti-inflammation of alginate-curcumin micelle for ulcerative colitis treatment. Colloids Surf. B Biointerfaces.

[5] Shi H., Wang D., Chen W., Li Y., Si G., Yang T. (2022). Quality of evidence supporting the role of supplement curcumin for the treatment of ulcerative colitis: An overview of systematic reviews. Gastroenterol. Res. Pract..

[6] Jiang M., Gan Y., Li Y., Qi Y., Zhou Z., Fang X., Jiao J., Han X., Gao W., Zhao J. (2023). Protein-polysaccharide-based delivery systems for enhancing the bioavailability of curcumin: A review. Int. J. Biol. Macromol..

[7] Zhang X., Ma Y., Ma L., Zu M., Song H., Xiao B. (2019). Oral administration of chondroitin sulfate-functionalized nanoparticles for colonic macrophage-targeted drug delivery. Carbohydr. Polym..

[8] Ye N., Zhao P., Ayue S., Qi S., Ye Y., He H., Dai L., Luo R., Chang D., Gao F. (2023). Folic acid-modified lactoferrin nanoparticles coated with a laminarin layer loaded curcumin with dual-targeting for ulcerative colitis treatment. Int. J. Biol. Macromol..

[9] Wu M., Ping H., Wang K., Ding H., Zhang M., Yang Z., Du Q. (2023). Oral delivery of pectin-chitosan hydrogels entrapping macrophage-targeted curcumin-loaded liposomes for the treatment of ulcerative colitis. Int. J. Pharm..

[10] Li X.-L., Yang B.-Q., Liu W.-J., Tao H., Zhang B. (2024). Curcumin loaded high internal phase emulsions: Formulation and potential to alleviate DSS-induced ulcerative colitis. Food Biosci..

[11] Xu J., Yan S., Qi B., Jiang L. (2025). New insights into the cross-linking mechanism of soybean protein-based double dynamic cross-linking hydrogels for the controlled delivery of curcumin. Food Res. Int..

[12] Zhang Q., Liu Y., Yang G., Kong H., Guo L., Wei G. (2023). Recent advances in protein hydrogels: From design, structural and functional regulations to healthcare applications. Chem. Eng. J..

[13] Milenkova S., Zahariev N., Ambrus R., Pilicheva B., Marudova M. (2023). A study on the stoichiometry of casein/chitosan gel complexes as a delivery system for quercetin. Appl. Sci..

[14] Peng H., Chen S., Luo M., Ning F., Zhu X., Xiong H. (2016). Preparation and self-assembly mechanism of bovine serum albumin–citrus peel pectin conjugated hydrogel: A potential delivery system for vitamin C. J. Agric. Food Chem..

[15] Liu Z., Liu C., Sun X., Zhang S., Yuan Y., Wang D., Xu Y. (2020). Fabrication and characterization of cold-gelation whey protein-chitosan complex hydrogels for the controlled release of curcumin. Food Hydrocoll..

[16] Liu F., Li R., Mao L., Gao Y. (2018). Ethanol-induced composite hydrogel based on propylene glycol alginate and zein: Formation, characterization and application. Food Chem..

[17] Yan J.-N., Wang Y.-Q., Li L., Zhang Z.-J., Gao L.-Y., Lai B., Wang C., Zhang L.-C., Wu H.-T. (2024). Scallop hydrolysates/κ-carrageenan hydrogels improve the alleviating effect of curcumin on DSS-induced colitis. J. Funct. Foods.

[18] Du Y.N., Xue S., Han J.R., Yan J.N., Shang W.H., Hong J.N., Wu H.T. (2020). Simultaneous extraction by acidic and saline solutions and characteristics of the lipids and proteins from large yellow croaker (*Pseudosciaena crocea*) roes. Food Chem..

[19] Du Y.-N., Han J.-R., Yin Z.-K., Yan J.-N., Jiang X.-Y., Wu H.-T. (2021). Conjugation of (–)-epigallocatechin-3-gallate and protein isolate from large yellow croaker (*Pseudosciaena crocea*) roe: Improvement of antioxidant activity and structural characteristics. J. Sci. Food Agric..

[20] Du Y.-N., Jia J., Yan J.-N., Xu S.-Q., Wang Y.-Q., Wu H.-T. (2024). Non-covalent interactions between large yellow croaker (*Pseudosciaena crocea*) roe protein isolates and curcumin: Implications for enhanced curcumin delivery. Food Biosci..

[21] Du Y.-N., Yan J.-N., Xu S.-Q., Wang Y.-Q., Wang X.-C., Wu H.-T. (2023). Formation and characteristics of curcumin-loaded binary gels formed from large yellow croaker (*Pseudosciaena crocea*) roe protein isolate and gellan gum. Food Chem..

[22] Yan J.-N., Du Y.-N., Jiang X.-Y., Xu S.-Q., Wu H.-T. (2022). Curcumin-loaded composite hydrogel based on scallop (*Patinopecten yessoensis*) male gonad hydrolysates and *κ*-carrageenan: Characterization and in vitro digestibility. Food Hydrocoll..

[23] Cao Y., Gao J., Zhang L., Qin N., Zhu B., Xia X. (2021). Jellyfish skin polysaccharides enhance intestinal barrier function and modulate the gut microbiota in mice with DSS-induced colitis. Food Funct..

[24] Wang C., Han Z., Wu Y., Lu X., Tang X., Xiao J., Li N. (2021). Enhancing stability and anti-inflammatory properties of curcumin in ulcerative colitis therapy using liposomes mediated colon-specific drug delivery system. Food Chem. Toxicol..

[25] Gong X., Cai W., Yang D., Wang W., Che H., Li H. (2025). Effect of the arabinogalactan from *Ixeris chinensis* (Thunb.) Nakai. attenuates DSS-induced colitis and accompanying depression-like behavior. Int. J. Biol. Macromol..

[26] Zhang L., Li F., Wang H., Chen B., Hua Y., An Z. (2025). Study on the ameliorative effects of hydroxypropyl betadex and betadex sulfobutyl ether sodium on acute ulcerative colitis induced by DSS in mice. Carbohydr. Polym. Technol. Appl..

[27] Pan J., Peng K., Ruan R., Liu Y., Cui X. (2025). Impact of anaerobic fermentation liquid on bok choy and mechanism of combined vitamin C from bok choy and allicin in treatment of DSS colitis. Foods.

[28] Salem M.B., Elzallat M., Mostafa Mohammed D., Hammam O.A., Tamim A., Abdel-Wareth M., Hassan M. (2024). Helix pomatia mucin alleviates DSS-induced colitis in mice: Unraveling the cross talk between microbiota and intestinal chemokine. Heliyon.

[29] Zhang L., Liu X., Xu M., Cheng X., Li N., Xu H., Feng Y., Guan T., Xiao L. (2025). *Patrinia scabiosaefolia* L. modulates the intestinal microecology to treat DSS-induced ulcerative colitis: UHPLC-OE-MS/MS, network pharmacology, and experimental validation. Foods.

[30] Zhang S., Li C. (2024). A curcumin-loaded biopolymeric nanocomposite alleviates dextran sulfate sodium induced ulcerative colitis via suppression of inflammation and oxidative stress. Int. J. Biol. Macromol..

[31] Zhang C., Wang X., Xiao M., Ma J., Qu Y., Zou L., Zhang J. (2022). Nano-in-micro alginate/chitosan hydrogel via electrospray technology for orally curcumin delivery to effectively alleviate ulcerative colitis. Mater. Des..

[32] Machado M., Silva S., Costa E. (2024). Chapter 15—Uses of gellan gum for nutrient delivery. Application of Gellan Gum as a Biomedical Polymer.

[33] Mundlia J., Munish A., Kumar P. (2021). Enhanced biological activity of polyphenols on conjugation with gellan gum. Int. J. Polym. Mater. Polym. Biomater..

[34] Xu S.-Q., Liu H.-X., Yan J.-N., Wang C., Lai B., Wu H.-T. (2024). Interaction mechanism and binding mode between different polyphenols and gellan gum. Food Hydrocoll..

[35] Ma P., Si X., Chen Q., Ma L., Hou M., Xu Z., Kang Y., Wang J., Xiao B. (2019). Oral drug delivery systems for ulcerative colitis therapy: A comparative study with microparticles and nanoparticles. Curr. Cancer Drug Targets.

[36] Pagano E., Romano B., Iannotti F.A., Parisi O.A., D’Armiento M., Pignatiello S., Coretti L., Lucafò M., Venneri T., Stocco G. (2019). The non-euphoric phytocannabinoid cannabidivarin counteracts intestinal inflammation in mice and cytokine expression in biopsies from UC pediatric patients. Pharmacol. Res..

[37] Song J.-L., Qian Y., Li G.-J., Zhao X. (2013). Anti-inflammatory effects of kudingcha methanol extract (Ilex kudingcha C.J. Tseng) in dextran sulfate sodium-induced ulcerative colitis. Mol. Med. Rep..

[38] Peng Y., Zhu J., Li Y., Yue X., Peng Y. (2024). Almond polysaccharides inhibit DSS-induced inflammatory response in ulcerative colitis mice through NF-κB pathway. Int. J. Biol. Macromol..

[39] Camacho-Barquero L., Villegas I., Sánchez-Calvo J.M., Talero E., Sánchez-Fidalgo S., Motilva V., de la Alarcón Lastra C. (2007). Curcumin, a Curcuma longa constituent, acts on MAPK p38 pathway modulating COX-2 and iNOS expression in chronic experimental colitis. Int. Immunopharmacol..

[40] Hu Y., Zhang S., Wen Z., Fu H., Hu J., Ye X., Kang L., Li X., Yang X. (2022). Oral delivery of curcumin via multi-bioresponsive polyvinyl alcohol and guar gum based double-membrane microgels for ulcerative colitis therapy. Int. J. Biol. Macromol..

[41] Mudter J., Neurath M.F. (2007). II-6 signaling in inflammatory bowel disease: Pathophysiological role and clinical relevance. Inflamm. Bowel Dis..

[42] Beck P.L., Wallace J.L. (1997). Cytokines in inflammatory bowel disease. Mediat. Inflamm..

[43] Mosmann T.R., Sad S. (1996). The expanding universe of T-cell subsets: Th1, Th2 and more. Immunol. Today.

[44] Walker A.W., Sanderson J.D., Churcher C., Parkes G.C., Hudspith B.N., Rayment N., Brostoff J., Parkhill J., Dougan G., Petrovska L. (2011). High-throughput clone library analysis of the mucosa-associated microbiota reveals dysbiosis and differences between inflamed and non-inflamed regions of the intestine in inflammatory bowel disease. BMC Microbiol..

[45] Yang X., Li S., Wang C., Lou Y., Xia X., Xu H. (2021). Whole and polysaccharide powdered Sporisorium reilianum improves DSS-induced colitis in BALB/c mice by modulating gut microbiota. J. Funct. Foods.

[46] Li Y., Pan H., Liu J.-X., Li T., Liu S., Shi W., Sun C., Fan M., Xue L., Wang Y. (2019). I-Arabinose inhibits colitis by modulating gut microbiota in mice. J. Agric. Food Chem..

[47] Wang N., Wu T., Du D., Mei J., Luo H., Liu Z., Saleemi M.K., Zhang R., Chang C., Mehmood M.A. (2022). Transcriptome and gut microbiota profiling revealed the protective effect of tibetan tea on ulcerative colitis in mice. Front. Microbiol..

[48] Shao J., Li Z., Gao Y., Zhao K., Lin M., Li Y., Wang S., Liu Y., Chen L. (2021). Construction of a “Bacteria-Metabolites” co-expression network to clarify the anti–ulcerative colitis effect of flavonoids of sophora flavescens aiton by regulating the “Host–Microbe” interaction. Front. Pharmacol..

[49] Mukhopadhya I., Hansen R., El-Omar E.M., Hold G.L. (2012). IBD—What role do Proteobacteria play?. Nat. Rev. Gastroenterol. Hepatol..

[50] Goldstein E.J.C., Citron D.M., Peraino V.A., Cross S.A. (2003). *Desulfovibrio desulfuricans* bacteremia and review of human *Desulfovibrio* infections. J. Clin. Microbiol..

[51] Latham E.A., Pinchak W.E., Trachsel J., Allen H.K., Callaway T.R., Nisbet D.J., Anderson R.C. (2019). Paenibacillus 79R4, a potential rumen probiotic to enhance nitrite detoxification and methane mitigation in nitrate-treated ruminants. Sci. Total Environ..

[52] Latham E.A., Pinchak W.E., Trachsel J., Allen H.K., Callaway T.R., Nisbet D.J., Anderson R.C. (2018). Isolation, characterization and strain selection of a Paenibacillus species for use as a probiotic to aid in ruminal methane mitigation, nitrate/nitrite detoxification and food safety. Bioresour. Technol..

